# Exploring the social context of insomnia: a thematic content analysis of the lived experiences of insomnia of Latinx women and men

**DOI:** 10.3389/frsle.2024.1456045

**Published:** 2024-09-11

**Authors:** Luciana Giorgio Cosenzo, Darwin Arias, Carmela Alcántara

**Affiliations:** ^1^The University of Alabama, School of Social Work, Tuscaloosa, AL, Untied States; ^2^Columbia University, School of Social Work, New York, NY, Untied States

**Keywords:** sleep health, health disparities, social ties, Latinxs, gender differences

## Abstract

**Introduction:**

Although social ties have detrimental and beneficial effects on sleep health, the social processes through which social ties affect sleep remain understudied, particularly among Latinx adults. These processes include social support, social stress, social control, personal control, and symbolic meaning. Importantly, some studies suggest there are gender differences in how social ties influence Latinx health. This study explores how Latinx women and men with insomnia describe the social processes through which social ties shape their lived experience of insomnia.

**Methods:**

Qualitative data from six focus groups conducted in 2017 were analyzed using thematic content analysis. These focus groups were comprised of 35 Spanish- and English-speaking Latinx adults with insomnia. Participants were majority women (62.86%), had a mean age of 65.43 years (SD = 12.63), and an average insomnia severity score of 20.6 (SD = 3.44). Major and minor themes were deductively and inductively identified in the transcripts. These themes were compared between Latinx women and men. The methods and results of this study are reported using the Consolidated Criteria for Qualitative Research (COREQ).

**Results:**

The influence of social processes on the lived experience of insomnia centered on three major themes. The first theme identified social stress and social control as precipitating and perpetuating factors of insomnia. The second theme related to how social support, through receiving advice from others, shaped participants' coping behaviors. Lastly, participants described their insomnia symptoms as negatively affecting their social relationships through social conflict. Latina women attributed their insomnia to the symbolic meaning of their gender identities, while men did not.

**Discussion:**

These findings suggest that among Latinx adults, it may be important for psychological treatments for insomnia to address the ways in which social processes contribute to insomnia symptoms. Additionally, Latina women's discussion of the influence of gender identities on their insomnia symptoms highlights the need to tailor psychological treatments for Latina women that acknowledge and address the challenges presented by traditional gender roles and expectations. Future studies should investigate the potential reciprocal relationship between social processes, mainly social stress and social control, and insomnia symptoms.

## Introduction

Insomnia, a psychiatric disorder characterized by difficulty initiating or maintaining sleep, or waking up too early accompanied by psychological distress (American Psychiatric Association, 2013), is the most common sleep disorder in the United States (US) affecting approximately one in three adults (Olfson et al., 2018). Latinx adults have experienced more accelerated increases in the prevalence of this disorder between 2002 and 2012 (16.6%−19.3%) than any other racial/ethnic group in the US, yet, insomnia remains understudied and underdiagnosed in this population (Loredo et al., 2010; Ford et al., 2015). The accelerated increase in the prevalence of insomnia among Latinx adults is particularly alarming considering the adverse health outcomes associated with this disorder including an increased risk of depression and anxiety (Alvaro et al., 2013), diabetes (Cespedes et al., 2016; Khan and Aouad, 2017), and cardiovascular disease (Javaheri and Redline, 2017; Khan and Aouad, 2017), which already affect a large percentage of this population (Alegría et al., 2008; Daviglus et al., 2012; Schneiderman et al., 2014). Recent studies have emphasized the influential effects of social ties on sleep health and the development of sleep disorders (Kent de Grey et al., 2018; Gordon et al., 2021). Although the exact mechanisms through which social ties impact sleep health remain elusive, conceptual models linking social ties to health outcomes suggest social processes, the active components of social ties, may shape health behaviors which, in turn, impact health (Umberson et al., 2010). These social processes include social support, social stress, social control, personal control, and symbolic meaning (Umberson et al., 2010). A burgeoning body of research has begun to document the associations between these social processes and sleep health, particularly social support and social stress and sleep quality, however, investigations of other social processes remain in their infancy.

Studies have examined the associations of social support and social stress on sleep in different social ties domains, such as romantic ties, family ties and friend ties. The effects of relationship quality on sleep health have been well documented among married couples (Troxel et al., 2010, 2017). Troxel et al. (2009, 2010) demonstrated that good quality marital relationships, measured through marital satisfaction and happiness, were significantly associated with better sleep quality and longer sleep duration in large samples of multi-ethnic women. A meta-analysis of 61 studies conducted among majority non-Latinx White samples demonstrated that social support from various types of relationships including family, friend, and romantic relationships was significantly associated with improved sleep outcomes including increased sleep quality and duration, and decreased sleep disturbances and insomnia symptoms (Kent de Grey et al., 2018). Social stress, although comparatively less studied in sleep health research, has been consistently associated with poor sleep quality (Chung, 2017; Stafford et al., 2017) and an increased risk of experiencing insomnia symptoms (Ailshire and Burgard, 2012). These findings suggest that social support and social stress from different social tie domains can either promote sleep health or be risk factors for poor sleep health, including insomnia symptoms.

Social control, a social process which refers to how the environment, norms, values, and beliefs created by one's social ties facilitate or prohibit individual health behaviors (Umberson et al., 2010), is theorized to affect insomnia through enforcing poor sleep habits among individuals who sleep in shared environments (Rogojanski et al., 2013). For example, romantic partners exert social control on each other through creating and enforcing daytime and bedtime routines (Rogojanski et al., 2013). These routines may increase or decrease the risk of experiencing insomnia symptoms depending on the compatibility of sleep-wake cycles between partners. For instance, sharing a bedtime that is compatible with both partners' sleep-wake cycles could help establish regular bedtimes, a healthy sleep habit that may reduce the risk of experiencing insomnia symptoms (Bootzin and Epstein, 2000; Morin, 2011). Conversely, the social control exerted in the relationship causing one person to go to bed at a time incompatible with their natural sleep-wake pattern may perpetuate and possibly exacerbate insomnia symptoms in that person (Bootzin and Epstein, 2000; Morin, 2011). These mismatches in bedtimes have also been associated with increased conflict in romantic relationships, an additional stressor which could precipitate insomnia symptoms.

Studies examining the association between personal control, a social process defined as one's belief that one is able to engage in goal directed actions to directly impact one's health outcomes (Umberson et al., 2010; Mirowsky and Ross, 2017) and insomnia symptoms have highlighted the ways in which this social process can be detrimental and beneficial to sleep quality. On one hand, individuals experiencing insomnia symptoms may exercise personal control by engaging in behaviors that they believe will induce sleep. For example, people with insomnia may decide to lay in bed before they feel sleepy to try to initiate sleep (Harvey, 2002; Espie et al., 2006). However, effortful attempts to induce sleep are often counterproductive, promoting psychological and physiologic arousal instead of rest (Harvey, 2002; Espie et al., 2006). A study conducted among people with chronic insomnia found that increased perceived personal control, measured through one's belief in one's ability to fall and stay asleep, was associated with increased sleep-anxiety, a common symptom that co-occurs with insomnia (Vincent et al., 2004). On the other hand, studies have indicated that increased personal control, operationalized as confidence in one's ability to engage in behaviors that promote sleep (i.e., relaxing before bed), is associated with lower insomnia symptoms (Shirota et al., 1998; Bluestein et al., 2010). These studies suggest social processes may impact insomnia symptoms by influencing internal factors, such as one's belief in one's ability to sleep or engage in healthy sleep habits.

Although the association between symbolic meaning and sleep behaviors was initially theorized in the early 1990's (Taylor, 1993), very few sleep health investigations have examined this relationship. This process is more difficult to measure than other social processes because it refers to the perceived social status ascribed to specific behaviors within a social context (Umberson et al., 2010). This social status may shift over time and be applied differently by individuals with different social identities. One qualitative study exploring men's attitudes toward sleep found that most men described sleep as essential to performing their social roles, but they also prioritized performing those social roles over sleep duration or quality (Meadows et al., 2008). Separately, a quantitative study conducted among women and men examining the relationship between masculinity and sleep found that men who were described to sleep “a little” vs. men who were described to sleep “a lot” in vignettes were rated as more masculine (Warren and Campbell, 2021). In the same study, those who were asked to design male characters who were stereotypically very masculine described their characters as sleeping significantly fewer minutes than those who were asked to design characters who were stereotypically not masculine (Warren and Campbell, 2021). These findings suggest that healthy sleep may not be a desirable behavior among men who ascribe to masculine ideals (Warren and Campbell, 2021). To date, women's views on sleep and its impact on their social roles or status have not been examined.

It is important to note that the relationship between social processes and health may not be the same across cultural groups. For example, among Latinx adults, the relationship between social support and sleep health may have dualistic, meaning positive and negative effects on sleep health, whereas among non-Latinx White adults social support has been consistently associated with positive sleep health (Kent de Grey et al., 2018). The Latinx cultural orientation of convivial collectivism which emphasizes interdependence among individuals to promote the wellbeing of the group, particularly within families (Campos et al., 2014), contrasts the European-American cultural orientation of individualism which emphasizes independence and prioritizes one's individual wellbeing (Chun et al., 2006; Campos et al., 2014; Campos and Kim, 2017). Convivial collectivism may lead Latinx adults to experience social stress through increasing feelings of social obligation toward others (Chun et al., 2006; Campos and Kim, 2017). In a qualitative study by Viruell-Fuentes (2007) exploring the influence of social ties on Latinx health among first- and second-generation Mexican women, first-generation immigrant women described having benefited from family social support in their transition from Mexico to the US but felt burdened by the obligation to reciprocate the support while having limited resources (Viruell-Fuentes, 2007). Importantly, quantitative studies on social ties and Latinx health have found gender differences in the association between social support and varying health outcomes (Alcántara et al., 2015; Torres et al., 2018). While social support was consistently associated with positive health outcomes among men, among women increased social support was associated with increased odds of experiencing depressive symptoms (Torres et al., 2016), smoking (Alcántara et al., 2015), and increased levels of inflammatory biomarkers (Torres et al., 2018). These gender differences have been underexplored in sleep health studies.

Differences in the types of social stress, social control, and personal control experienced by Latinx adults may also exist. In a qualitative study, second-generation Mexican women described their family, friend, and neighborhood social ties as allowing them to explore Mexican culture and develop their identities, but also exposing them to racial/ethnic discrimination (Viruell-Fuentes, 2007). The experience of racial/ethnic discrimination, a type of social stress that non-Latinx Whites may not experience, may negatively impact Latinxs' lived experience of insomnia. Additionally, social control and personal control may also be experienced differently in this ethnic group because Latinx cultural values center family as referents of attitudes and behaviors (Sabogal et al., 1987) throughout the life course whereas among non-Latinx White adults, familial influence is largely limited to childhood and adolescence (Freeberg and Stein, 1996). Within-group examinations of how social process impact insomnia could elucidate these differences.

Although there is enough evidence to suggest there is a relationship among social support, social stress, social control, personal control, symbolic meaning and insomnia symptoms, further research is needed to explore how these social processes are understood and experienced by those suffering from insomnia (Umberson et al., 2010; Kent de Grey et al., 2018; Gordon et al., 2021). Most sleep health studies have utilized quantitative methods to test the relationship between different social processes and insomnia symptoms; however, qualitative methods may provide a richer exploration of these interacting social processes through participants' descriptions (Araújo et al., 2017; Creswell and Clark, 2017). Conducting qualitative studies on people's lived experiences of insomnia and the social processes that affect that lived experience could generate a deeper understanding of the complex risk and protective social factors of this psychiatric disorder. Further, the associations between social processes and sleep health have been examined in majority non-Latinx White samples, with the exception of one study that reported results stratified by ethnicity (Troxel et al., 2009). This limits the generalizability of the findings to populations with similar conceptualizations of the role of social processes in shaping one's values, beliefs, and behaviors.

This study used thematic content analysis (Braun and Clarke, 2006; Vaismoradi et al., 2013) to explore how Latinx adults describe the social processes through which social ties affect their lived experience of insomnia. Because widely referenced models of insomnia describe the pathogenesis of this disorder as a combination of predisposing, precipitating, and perpetuating factors (Borbély, 1982; Spielman et al., 1987), the thematic content analysis highlighted descriptions of how social processes contribute to these factors. Predisposing factors refer to an individual's characteristics or traits that increase their risk of experiencing insomnia (Spielman et al., 1987). Precipitating factors are often described as stressful events or circumstances that trigger the onset of insomnia symptoms (Spielman et al., 1987). Perpetuating factors are habitual behaviors that maintain the experience of insomnia symptoms even after precipitating factors subside (Spielman et al., 1987). Additionally, this study explored differences and similarities in how Latinx women and men describe the social processes that shape their lived experience of insomnia.

## Materials and methods

This analysis utilized focus group data collected from July through October 2017 as part of the CBT-I for Latinos (CLIO) study. CLIO aimed to explore culturally specific factors contributing to the initiation and maintenance of insomnia to inform the cultural adaptation of a psychological treatment for insomnia for Latinx adults and to examine the acceptability and feasibility of a digital treatment for insomnia. The consolidated criteria for reporting qualitative research (COREQ) (Tong et al., 2007) guided the reporting of the study methods and results in this analysis.

### Domain 1: research team and reflexivity

Focus groups were led by two to three bilingual and bicultural facilitators using a semi-structured interview guide. These facilitators were female immigrants from the Dominican Republic, Mexico, and Spain. All three facilitators had experience conducting sleep research and were actively engaged in primary data collection of sleep data in the Latinx community.

### Domain 2: study design

#### Theoretical framework

Focus group transcripts were coded and interpreted using thematic content analysis (Braun and Clarke, 2006; Vaismoradi et al., 2013). Thematic content analysis is used to find, analyze, describe patterns in the form of themes across sources of qualitative data, and interpret the themes (Braun and Clarke, 2006).

#### Participant selection

Participants were recruited using convenience sampling methods at community centers, health fairs, and a primary health care clinic in New York via flyers and tabling events. Participants were also drawn from a repository of Latinx research volunteers. Before attending the focus groups, participants completed an eligibility survey. Participants were included in the study if they were 18 years of age or older, identified as Latino or Hispanic, spoke English, Spanish, or were bilingual, and reported experiencing clinically significant insomnia symptoms for three or more months based on Insomnia Severity Index (ISI) scores ≥15 (Morin et al., 2011). If participants were deemed unable to complete the study protocol due to cognitive impairment, severe medical or mental illness, or active substance use or were unable to attend the focus group sessions, they were excluded from the study. All participants provided informed consent before participating in this study. CLIO was approved by the University's Institutional Review Board (IRB AAAR1336).

Out of 95 people who completed the screening survey, 51 met the inclusion criteria, and 16 were excluded because they were no longer interested or unable to attend the focus group sessions. A total of six in-person focus groups (two in English and four in Spanish) were attended by nine English-speaking and 26 Spanish-speaking Latinx adults with chronic insomnia.

#### Setting

The CLIO study took place in New York City (NYC) where the Latinx population makes up approximately 29% of the total population (Greer et al., 2017). Over one third (37.14%) of participants were recruited from a primary care clinic in Washington Heights, where a large concentration of individuals from the Dominican Republic reside (26%−53% of the total Dominican population living in NYC) (Greer et al., 2017). The focus groups were conducted at a community health clinic located in Washington Heights, an ethnic enclave in NYC.

#### Data collection

Each focus group consisted of between four to nine participants. All focus group discussions included an explanation of the focus group goals, an exploration of the participants insomnia history and concluded with specific questions about CBT-I and its acceptability to participants. Each focus group was audio recorded using an encrypted device.

Focus group discussions had an average duration of 73.67 min with the longest focus group lasting 87 min and the shortest 60 min. After each focus group the audio recording was sent for professional transcription. A bilingual CLIO team member reviewed the transcripts for accuracy and de-identified them (i.e., removed participants' last names). These de-identified transcripts were used in this study. Although this study is based on secondary data analysis, theoretical saturation, defined as the point at which no new data is being collected on a particular topic (Morse, 1995; Saunders et al., 2018), is believed to have been attained with 35 participants.

### Domain 3: analysis and reporting

#### Data analysis

First, an initial review of the transcripts was conducted to further refine the preliminary codebook. Then, two bilingual coders (LGC and DA) coded the transcripts in their initial language of administration using the qualitative software program NVivo version 12 (QSR International Pty Ltd, 2018). To calibrate, the coders reviewed two transcripts independently (one English and one Spanish) and met to discuss coding decisions including new codes identified throughout the review. The coded segments of the remaining four transcripts were compared to examine the interrater agreement. An average of 94.75% agreement was achieved which indicates excellent interrater agreement (Gisev et al., 2013).

Although the entirety of the transcripts was reviewed, the responses to the questions “What factors would you say contributed most to the start of your insomnia? What factors would you say contribute to the continuation of your insomnia? Have specific factors related to your ethnicity, race, identity, socioeconomic status, language fluency, or neighborhood contributed to your insomnia? Why or why not?” were targeted during the coding process.

Excerpts were identified and grouped according to similarities and conceptual relationships using thematic content analysis (Vaismoradi et al., 2013). Codes and themes were guided by the research questions as well as developed inductively to allow for unexpected insights to emerge from the transcripts. The codebook used can be reviewed in the Supplementary material A. Coded excerpts were categorized by the gender of the respondent to explore potential differences in how Latinx women and men describe the social processes shaping their lived experience of insomnia.

#### Reporting

The participant's gender and focus group language of administration were indicated before representative quotations to conserve anonymity. Excerpts originally transcribed in Spanish were translated to English. Themes and patterns are described in detail throughout the results section differentiating between pre-specified themes and data-driven themes. A clear distinction between major and minor themes and subthemes was also included. Major themes reflect patterns in participant narratives that were discussed by multiple participants within each focus group and permeated across most focus group transcripts. Minor themes indicate topics that were mentioned by a few participants across multiple focus groups or represent a unique perspective generated in one or two focus groups.

## Results

Most participants were female (62.86%), on average participants were 65.43 years old (SD = 12.63) and had an average ISI score of 20.6 (SD = 3.44), which indicated moderate insomnia severity. Other participant characteristics are presented in Table 1. The influence of social processes on the lived experience of insomnia were categorized into three main themes: (1) social processes as predisposing, precipitating, and perpetuating factors of insomnia, (2) social processes' impact on coping with insomnia, and (3) insomnia symptoms' effects on social relationships. Within each theme, major and minor subthemes were identified. Participant quotations for each theme and subtheme are represented in Table 2. Across all themes, family social ties followed by spouses/romantic partners were mentioned more frequently in coded segments compared to other social tie domains.

**Table 1:** Sociodemographic and insomnia symptom characteristics of Latinxs participating in CLIO (*n* = 35).

	**All (*****n*** = **35)**		**Women (*****n*** = **22, 62.86%)**		**Men (*****n*** = **13, 37.14%)**	
	***N*** **/M**	**%/SD**	***N*** **/M**	**%/SD**	***N*** **/M**	**%/SD**
Age, y	65.43	12.63	64.86	12.70	66.38	12.96
**Nativity status**						
Immigrant	27	77.14%	18	81.82	9	69.23
U.S.-born	8	22.86%	4	18.18	4	30.77
**Country of origin or heritage**						
Chile	1	2.86	0	0	1	7.69
Colombia	2	5.71	1	4.55	1	7.69
Cuba	1	2.86	0	0	1	7.69
Dominican Republic	17	48.57	14	63.64	3	23.08
Ecuador	1	2.86	0	0	1	7.69
Italy	1	2.86	1	4.55	0	0
Mexico	4	11.43	1	4.55	3	23.08
Peru	1	2.86	0	0	1	7.69
Puerto Rico	6	17.14	4	18.18	2	15.38
Venezuela	1	2.86	1	4.55	0	0
**Language of focus group**						
English	9	25.71	2	9.09	7	53.85
Spanish	26	74.29	20	90.91	6	46.15
**Language fluency**						
Spanish only	14	42.42%	11	52.38	3	25.00
English only	3	9.09%	2	9.52	1	8.33
Bilingual	16	48.48%	8	38.10	8	66.67
Missing	2	5.70%				
**Educational attainment**						
Less than High School	6	17.14	5	22.73	1	7.69
High school diploma or GED	11	31.43	8	36.36	3	23.08
Trade School/Vocational School	2	5.71	1	4.55	1	7.69
Some College	6	17.14	4	18.18	2	15.38
College graduate or Graduate degree	10	28.57	4	18.18	6	46.15
Insomnia Severity Index Score (min 15, max 27)	20.6	3.44	20.32	3.68	21.08	3.07

**Table 2:** Examples of quotations of subthemes across three major themes demonstrating the influence of social processes on the lived experience of insomnia among Latinx adults.

**Major themes**	**Social processes**	**Subthemes**	**Influential social tie domains**	**Illustrative quotes**
Major Theme 1: Social processes as predisposing, precipitating, and perpetuating factors of insomnia	Social stress	Worry and rumination about other's problems	Family	F2(ES): In my case particularly, I know that first of all, I cannot separate the negative things that happen to me during the day, I can't dissociate with them at night. So, for example, at this moment I am having a lot of issues with my son. He is in Colombia, but I have problems with him. So, I spend all night, eh, thinking about what I'm going to say to him when I go back to Colombia, and I write it…
			Work	M1(ES): Um, for me at least, I started an anxiety, depression, at work. I mean, I don't know when it [insomnia] came, but it was due to work. And I think this is how it happened, because at work I had to—the supervisor would say, “Np, you have to go this other thing.” And I would say, “But how? I am doing something else and now I have to do something else?” “No, no you have to do to this.” And you know, where a captain rules a sailor has no sway. So, I couldn't contradict him. So, I would stop doing one thing and the next day I would do the same thing, the same work. A lot of tasks were pending and- and my head was like this that I couldn't take it anymore. And I would go to that, I would go home with all the burden of work and the problems at home, on top of the problems at work, I think little by little, that eat away at me until, like a glass filling up with water until it overflows. And that's when it seems that anxiety of the depression gripped me and form there I started [with insomnia symptoms]
		Loneliness	Family/spouse	M1 (EN): And for me a big one has to do with, uh, loneliness, um I recently got divorced three years ago, and, um, that's a big issue for me, not being with my wife, my kids are in North Carolina
			Family	M6 (ES): In general, all the things affect me, for example mid-life crisis… But in particular de fact of… of being separated from family members of so much time…The lack of company… the fact of being alone
			Spouse/friends	F3(EN): I mean, this thing of waking up at 2:00, 3:00 in the morning, it's, like you know my husband is sleeping, here I am, you know, reading a book or the TV, the computer, 7:00 I'm doing my laundry, 8:00 I'm in the supermarket… everyone else is sleep, you know? All my friends, “Oh, I'm sleeping,” you know?
		Ethnic discrimination	Others	F5(ES): Because one is alert to what else he [President Trump] will put into effect. I mean, my family, thank God, my daughter with whom I live with is a citizen, her children were born here. The other one is in the processes of becoming a resident and I am a legal resident. I mean, directly it doesn't affect me, but with all the new things he says, and he says he is going to do and that for Dominican their status will change, how do you call it? The residence to a visitor's visa…. It keeps me awake the attitude of the president
	Social control	Others impacting bedtime routines and sleep habits	Family	F4(EN): I think that [the insomnia symptoms] started because, actually, all my family, it seems like they have insomnia, too. They were always up all night, and I was the only one who was going to school, so then I kind of had that sleep schedule that there is no sleep. [So], now I try to fall asleep, I lay there, there's so many thoughts I can't turn off my brain off, I keep thinking
			Neighbor	F1(EN): I actually have a neighbor that works at night, I believe he works in a club, so his hours are just all over the place and when I finally am getting to sleep, about five or so, that's when he's coming in and he's making noise and he's, you know, throwing his shoes on the floor, and- so that's another- another factor, to, you know, when I'm trying to get back to sleep, and he's just starting his day pretty much and he's just noisy- so that contributes to- to it
	Social stress	Acculturation stress	Family	F5(ES): For me, it's the change in culture once one is older, it's- there are many customs that I can't adapt to. And there are behaviors that I hear, “How can this be happening in my family?”. So, that, I think that those are the things that contribute to one staying awake
	Personal control	Poor sleep hygiene	Self	F3(ES): But I think that influences, like she said, the bad habits. An example, I eat late. I eat late. And sometimes, when I eat something heavy. “This, this night call it quits, there will be no sleep.” And I know, I know
	Symbolic meaning	Womanhood/motherhood	Family	Speaking about what gets in the way of sleep… • F3(ES): It's because as a woman, we were taught since we were young to do everything at home. When the man can wash dishes, he can help wash clothes, he can change a diaper and we let him sit there and what TV or go play sports. They are with friend instead of helping us. It's all of that
Major Theme 2: Social processes' impact on coping with insomnia	Social support	Receiving helpful and unhelpful social support	Family	• F4(EN): The very first thing my dad introduced me to was warm milk at night • F3 (ES): I have drunk teas. My mom says, “Drink linden tea” and that doesn't do anything for me
			Spouse	F1(EN): I would, too. I mean, it's- I just thought of my husband as he tries to get me to go to bed at a certain time and I feel like it's a parent telling a little child, you need to be right now, so…
	Social support	Pets serving as emotional support	Pets	F6 (ES): I take small naps and when I see I stay like that [awake] all night long, I drink tea or milk, and I continue the night like that. And I sip or I watch TV, or I play with the baby [the dog] and like that. The dog helped me feel calm
	Personal control	Poor and healthy sleep hygiene behaviors	Self	M2 (EN): Actually, I also do the same, like I'm watching TV, I put the sleep, uh, thing and that's how I fall asleep usually, but recently I've been trying to sleep more with the lights off and everything
Major Theme 3: Effects of insomnia on social relationships	Social stress/social support/social control	Interpersonal conflicts	Spouse	F3 (ES): I started to have problems with my husband. Not serious problems that, let's say, affected our relationship as a couple in that way, but yes of feeling a certain discomfort, because, I don't know, but most of you know all of the things that come along with menopausal symptoms, which is what I had in the moment. Those hot flashes started that I don't have anymore. I don't know, a lot of situations that those hormonal changes bring about. So, sometimes our partners don't understand the situation and so what they see is, “Ay, why do you have to go to bed so late? You really go around in circles”
		Not wanting to disturb others' sleep	Spouse	F1 (EN):I don't stay in the bed, I actually get up and go to the bathroom, so I don't disturb my husband, and like you said a learned behavior…
			Spouse/romantic partner	M2 (ES): When [my partner] sees that I am awake, they say “Turn the TV on, distract yourself,” they say. Uh, that's what I do, but I feel uncomfortable that I am robbing them of their sleep. And this isn't something that is one month, or one week, this has been going on awhile. The next day, I see that the other person leaves, they wake up, do their normal routine, and I see they are tired. Meaning, because they support me, they are being affected

### Major theme 1: social processes as predisposing, precipitating, and perpetuating factors of insomnia

Although precipitating and perpetuating factors were asked about separately during the focus group interviews, participants did not demarcate their experiences in this manner. Therefore, descriptions of precipitating and perpetuating factors are described in tandem. Because predisposing factors were spontaneously discussed in the focus groups, they were included in these results. Overall, social stress was identified as the main social process contributing to the precipitation and perpetuation of insomnia symptoms. The major themes described below are types of social stress and social control that influenced participants' insomnia symptoms. Personal control and symbolic meaning were minor themes. Social support was not discussed when describing these factors.

#### Major subthemes

##### Social stress: worry and rumination about others' problems

One of the most prominent themes was the influence of social stress experienced within family ties on insomnia symptoms. Several participants attributed the precipitation of their insomnia symptoms to illnesses or deaths of loved ones, particularly children and close relatives. These impactful events were also referenced when participants described the perpetuating factors of their insomnia. They described not being able to fall asleep because they ruminated on these events. An English-speaking woman described her experience having difficulty maintaining sleep:

I've lost two brothers in the past five years, so it's just waking up and they all were tragic, they all… passed away tragically, so it's like I can't believe this happened, you just start going through memories and the whole situation, so- and I- I don't stay in bed… sometimes I just go through life experiences and, you know, memories and it just, you know, keeps going and going and before I know it hours have passed.

Participants also described how their sleep was affected by their constant worry about others' wellbeing, particularly family members in and outside the United States. One Spanish-speaking woman summarized the discussion stating, “Each child that you have [that has a problem] or family member that has a problem, it's also your [problem]. And unfortunately, we dwell too much on those problems.” Participants seldom described their worry about not being able to fall asleep, which is a common perpetuating factor of insomnia (Harvey, 2002; Carney et al., 2006), as contributing to their insomnia symptoms.

##### Social stress: loneliness

Another social stressor described as a precipitating and perpetuating factor was loneliness. Several participants, particularly men, attributed their insomnia to being separated from their spouses and families. These separations were due to divorce/break up and migration to the United States. One Spanish-speaking man stated:

And also, the fact of family separation. The lack of affection, the lack of… living alone in a place, in a room or an apartment, no matter how luxurious it is, one is alone, and one gets home from work and goes. And at night it is when it [the separation] is most evident, the problem has more weight.

##### Social stress: ethnic discrimination

Participants described anti-immigrant sentiment in the media and ethnic discrimination as a source of ongoing social stress impeding good quality sleep. Some participants made specific references to the anti-immigrant rhetoric used by the 2016–2020 Presidential Administration of the United States of America (Finley and Esposito, 2020) as a source of stress for the wellbeing of their families, their communities, and themselves. Others described difficulty securing high-paying employment and financial stability despite being well educated because they are immigrants. One Spanish-speaking man stated:

The thing is that we don't earn like the Whites earn, you know? An example, I have a degree in business administration. I cannot practice my profession here, because I would have to go to college again to study. And imagine, me at 63 years old going to college, with a family to support.

##### Social control: others dictating bedtime routines

Social control was described as a predisposing, precipitating, and perpetuating factor of insomnia by establishing undesirable sleep patterns among participants. These sleep patterns tended to be determined by parents during childhood (predisposing factor) or by spouses/romantic partners' sleep habits (precipitating and perpetuating factors). One English-speaking woman described how her insomnia may have developed when her partner's sleep schedule started to conflict with her schedule stating:

And another part was I had a significant other who worked the night shift, so it kind of worked out that I would fall asleep during the day with him, and I would be up at night and it kind of worked out that way. But then, afterwards, I need to have a regular schedule, you know? I'd go to my appointments or- and now I have to because I have my home attendant be up at a certain time, so there's a few things that's contributing to the insomnia.

##### Minor subthemes

A minor theme related to social stress due to acculturation stress was discussed at great length in two focus groups. Several participants described feeling like outsiders in their families and communities due to differing views on cultural values, such as *respeto* [respect], which encourages a formal politeness and regard for authority, especially of elders, and *familismo* [familism], which emphasizes the importance of warm, interdependent relationships within the family (Campos and Kim, 2017). They stated that their concerns over what they viewed as a deterioration of values within their families contributed to the worries that kept them up at night. Two other social processes—personal control and symbolic meaning—, were also described as predisposing, precipitating, or perpetuating factors of insomnia. Participants described their behaviors, such as using social media in bed, as directly contributing to their insomnia symptoms. The social process of symbolic meaning was invoked when women in a few focus groups described their experience of insomnia symptoms as part of womanhood and motherhood. They described caring for and worrying about their families as central to their social identities as women and mothers which often meant they spent fewer hours sleeping compared to other family members and experienced poor-quality sleep because they were preoccupied with concerns about their families.

### Major theme 2: Social processes' impact on coping with insomnia

Upon reviewing the transcripts, themes relating to how participants coped with their insomnia symptoms emerged. Social support was the major social process mentioned under this topic. Effective and ineffective efforts to cope with insomnia through personal control were also mentioned.

#### Major subthemes

##### Receiving helpful and unhelpful social support

Participants described attempts by spouses/romantic partners, family members, co-workers, and friends to support them through their insomnia. They characterized some social support as helpful such as receiving advice on natural products to use to induce sleep, even when the products did not work, or they were unwilling to try them. One Spanish-speaking woman stated, “A lot of people tell me to try natural products, whatever, I don't want to. I don't want to because I am afraid of becoming addicted to medication. I prefer to make tea, chamomile, linden, something natural and not to take medication. I don't want to.”

Other attempts at providing social support were perceived as overly controlling. For example, participants described their spouses' encouragement to go to sleep at a certain time as analogous to being told to go to bed by a parent. One Spanish-speaking women described a conversation in which her husband expresses concern for her lack of sleep and encourages her to go to bed even when she is not sleepy:

Because he argues with me “But woman, how are you—still?” He sees that is 2:00am, that it's 3:00am. Because he sees the clock and that I'm not in bed yet. So [he says] “You are going to get sick if you stay awake so long, you don't sleep.” [I answer] “But my love, if I'm not sleepy, what do you want me to do?”

Although not directly associated with insomnia symptoms, a few participants described friends and family members supporting their overall mental health to improve their sleep. One Spanish-speaking woman stated, “That is what my daughter-in-law and son say to me “Learn to say no.” Because I always try to please and please others.”

#### Minor subthemes

##### Pets serving as emotional support

One participant mentioned they benefited from the support and company of their dog. They stated that having the dog with them helped them feel calm and less lonely at night which helped them fall asleep.

##### Poor and healthy sleep practices

Several participants described the types of behaviors they engaged in to cope with their insomnia symptoms. It is worth noting that several participants described engaging in poor sleep practices such as watching TV in bed and eating heavy meals in the middle of the night to cope with their insomnia symptoms. Others described healthy sleep practices such as getting out of bed if they could not sleep and reading somewhere other than their bed until they got tired. Toward the end of the focus group, some participants shared their plans to create healthy sleep routines to improve their symptoms, such as establishing regular bedtimes and turning off the TV before going to sleep. These descriptions are indicative of personal control because participants attribute their insomnia symptoms to their personal actions.

### Major theme 3: effects of insomnia on social relationships

Patterns highlighting the influence of insomnia on social relationships became evident across all six focus groups. It is important to note that several of the participants' descriptions were prompted by a question about motivations for seeking insomnia treatment. Additionally, in these narratives, the overlapping influence of several social processes, mainly social support, social control, and social stress, became evident.

#### Major subthemes

##### Interpersonal conflicts

Participants described that their insomnia contributed to conflicts in their romantic relationships and created tense situations with their co-workers and family members. Several women described getting into arguments with their spouses in bed because of their sleep patterns and behaviors. One Spanish-speaking woman, who in addition to suffering from insomnia symptoms was experiencing menopausal symptoms, recounted an argument with her husband in the middle of the night:

One of these nights I got one of those hot flashes. Look, I took my sheet off, I took the comforter off, I took of my socks. I didn't wake up in the nude, I didn't take off my clothes by coincidence. And my husband says, “Don't move so much.” [I answered], “No, no, no, if you don't want me to move I'll leave the bedroom.” Because I don't know—we get in a bad mood because of this situation [insomnia].

Her story is an example of the ways in which social processes overlap. In this narrative the spouse contributed to the conflict by establishing social control of appropriate behavior in the bed (i.e., staying still) which was discordant with the participant's needs to feel comfortable in bed. Later in the narrative, the participant described how her spouse became more supportive once she explained her condition and did not expect her to stay in bed all night as she coped with her insomnia and menopause symptoms. This interaction between social processes was evident in other narratives.

##### Not wanting to disturb others

When describing motivations for seeking treatment for insomnia, participants cited their personal relationships because they felt insomnia was damaging them. In addition to the conflicts described above, participants expressed feeling guilty because they felt their insomnia was affecting their bed partner's sleep. For example, one Spanish-speaking man stated:

When [my partner] sees that I am awake, they say “Turn the TV on, distract yourself,” they say. Uh, that's what I do, but I feel uncomfortable that I am robbing them of their sleep. And this isn't something that is one month, or one week, this has been going on awhile. The next day, I see that the other person leaves, they wake up, do their normal routine, and I see they are tired. Meaning, because they support me, they are being affected.

##### Gender differences in themes

There were many similarities in the ways in which women and men described how social processes impacted their lived experience of insomnia. Women and men described influential social tie domains such as family and work ties with similar frequencies. Both genders also described stress associated with work and relationships with bosses and co-workers as contributing to their insomnia symptoms. However, women brought up how their spousal/romantic relationships and insomnia symptoms impacted each other more often than men. They also tended to describe social stress from caregiving for family members more often than men. Additionally, women attributed their insomnia symptoms, in part, to their social identities. They described that their identities as women and mothers meant they had to prioritize serving others above their need to obtain a good night's sleep.

## Discussion

This study explored how Latinx adults describe the social processes through which social ties impacted their lived experience of insomnia. The major subthemes identified in this study provided support to findings from previous studies examining social ties among Latinx adults suggesting social processes may serve as simultaneous sources of risk and protection against poor health outcomes (Viruell-Fuentes, 2006; Alcántara et al., 2015). For example, although some themes demonstrated that social processes impacted insomnia symptoms in the expected direction (i.e., social support was associated with improved sleep), some participants described their spouses/romantic partner's attempts to provide social support as sources of conflict and social stress. Highlights from the major subthemes are discussed in detail below. Taken together, these findings highlight the embeddedness of insomnia within the social context and highlight the importance of addressing the ways in which social relationships impact insomnia symptoms and vice versa as part of psychological treatments for this sleep disorder.

### Worry/rumination, loneliness, ethnic discrimination, and family sleep habits as precipitating and perpetuating factors of insomnia

While previous studies identified sleep-oriented worry and rumination as a precipitating and perpetuating factor of insomnia (Harvey, 2002; Carney et al., 2006, 2013), in this study, participants attributed their insomnia to ruminating about others such as painful memories of family and tense interpersonal interactions around bedtime. Participants also described worrying about their families', friends', and others' wellbeing. Notably, worries and ruminative thoughts were about family ties more often than other type of social ties. This finding is consistent with the central role of family ties emphasized in the Latinx cultural value of *familismo* (Campos et al., 2014; Campos and Kim, 2017).

Relatedly, loneliness, particularly due to separation from family due to migration, was mentioned as a precipitating and perpetuating factor of insomnia. Loneliness has been positively associated with insomnia symptoms among majority non-Latinx White samples (Hom et al., 2020). However, among Latinx adults, the cultural value of *familismo* may make the absence of family contact particularly detrimental for sleep. Additionally, some Latinx adults may not be able to contact or visit their families in their countries of origin for a variety of reasons (i.e., financial constraints), which may exacerbate loneliness compared to non-Latinx White adults. Future studies should explore the specific influence of transnational ties, migration, loneliness and insomnia symptoms among Latinx adults.

Another precipitating and perpetuating social factor identified in this study was ethnic discrimination. Participant descriptions confirmed quantitative studies conducted among population-based, nationally representative samples of Latinx adults demonstrating that increased ethnic discrimination is significantly associated with increased insomnia symptom severity (Alcántara et al., 2017, 2019). This mounting evidence underscores the need to reduce ethnic discrimination through community and policy-level interventions and support Latinx adults who experience this type of social stress to promote sleep health.

Participants described the ways in which the sleep habits of spouses/romantic partners, family members, neighbors, among others contributed to the initiation and maintenance of their insomnia symptoms. Previous studies have found similar influences within the family context (Fuligni et al., 2015; Kouros and El-Sheikh, 2017; Gunn and Eberhardt, 2019). One study conducted among non-Latinx White and African American families made up of a mother, a father, and one or more children demonstrated that the mother's objectively measured sleep duration and quality on a given night were significantly influenced by the child's and father's sleep duration and quality that night (Kouros and El-Sheikh, 2017). These results suggest that identifying and addressing the sleep habits of those living with individuals experiencing insomnia may be important to help improve insomnia symptoms among Latinx adults.

### Social support's impact on coping with insomnia

Participants tended to describe social support as being helpful when others recommended products and behaviors to improve sleep, such as drinking tea or meditating. Several participants described instances when others' attempts to provide support made them feel more distressed or helpless over their insomnia. Previous studies conducted among individuals with serious medical conditions, such as cancer and heart conditions, found similar sentiments when participants described unhelpful forms of social support from partners, friends, and family (Dakof and Taylor, 1990; Boutin-Foster, 2005). In these studies, participants described perceiving social support as unhelpful when their loved ones minimized the seriousness of their condition, provided unsolicited advice or advice without an implementation strategy, and criticized participants behaviors and attitudes toward their illness (Dakof and Taylor, 1990; Boutin-Foster, 2005). These descriptions parallel the experiences of participants in this study who expressed frustration at being told by spouses/romantic partners and others to go to bed at certain times or get more sleep without providing tangible ways to so. These narratives suggest that educating the loved ones of individuals suffering from insomnia on ways to provide helpful social support may be an important step in treating this sleep disorder.

### Negative effect of insomnia on social relationships

Participants attributed the tension and discomfort that they felt in their relationships with spouses/romantic partners, family members, and co-workers, in part, to their insomnia symptoms. These descriptions are corroborated by findings from quantitative studies conducted among romantic couples demonstrating that poor sleep quality was inversely associated with relationship satisfaction (Troxel et al., 2007, 2017). The association between insomnia and increased interpersonal conflict is logical given that people suffering from insomnia often express feeling irritable which may increase the likelihood of negative social interactions (Fernández-Mendoza et al., 2009; American Psychiatric Association, 2013). In most narratives shared, interpersonal conflicts arouse at bedtime when participants did not go to sleep with their spouses/romantic partners. The narratives followed a similar pattern of interaction between participants and their spouses/romantic partners. Mainly, participants' spouses/romantic partners often initiated the conversation expressing concern over the sleep problems of the participants, displaying social support. In their attempts to support their partners, they encouraged them to go to sleep at a certain time, exercising social control over their partners. However, the participants felt frustrated by their spouses/romantic partners and an argument ensued, indicating the presence of social stress. These narratives exemplify the interconnection among social control, social support, and social stress in the context of insomnia.

To completement and confirm these qualitative findings, future studies should explore how spouses/romantic partners and other loved ones of individuals with insomnia experience the effects of insomnia on their relationships. Additionally, given that social stress and insomnia symptoms may create a positive feedback loop which may maintain and exacerbate insomnia, studies should examine the reciprocal relationship between these factors. If these findings are confirmed, adapting psychological treatments for insomnia to incorporate elements of interpersonal therapy, a type of psychotherapy that addresses problem areas in interpersonal relationships as a means of supporting mental health (Weissman et al., 2008), may enhance the effectiveness of these treatments by addressing the social environments contributing to insomnia symptoms. Lastly, given that participants described the negative effects of insomnia on their relationships as one of the motivators for seeking insomnia treatment, future studies should test whether improvements in insomnia symptoms are associated with improved relationship satisfaction.

### Gender differences

As suggested by previous studies (Alcántara et al., 2015; Torres et al., 2016, 2018), gender differences in how social processes influenced the lived experience of insomnia were present in the focus group transcripts. The main differences between women and men's descriptions centered around caregiving as a precipitating and perpetuating factor of insomnia among women. Additionally, women cited their social identities as women and mothers as contributing to their insomnia symptoms. Specifically, participants mentioned receiving advice from loved ones to improve their overall mental health through self-care, specifically prioritizing themselves above others' needs. Even during the focus group discussions, participants interjected in one another's stories reinforcing this idea. These interactions reflect previous studies identifying the unequal distribution of household and caregiving responsibilities as a potential explanation for the increased prevalence of insomnia among women when compared to men (Yoshioka et al., 2012). Additionally, these descriptions coincide with studies conducted among Latina women demonstrating that increased endorsement of *marianismo*, which refers to the cultural gender role expectations of women to be nurturing, self-sacrificing, and subservient (Niemann, 2004; Nuñez et al., 2016), particularly regarding women's role as the primary caregivers of the family, were associated with poor mental and physical health outcomes (D'alonzo, 2012; Nuñez et al., 2016). However, literature examining the association between *marianismo* and health outcomes is very limited. Although the discussion on this topic was expansive, it was only present in an all-women focus group. Women may feel more comfortable expressing these sentiments among others who share similar life experiences due to their shared gender identity. Future studies may benefit from using mixed methods and gender-specific focus groups to explore whether the level of endorsement of cultural gender role expectations influence insomnia symptoms among Latinx women and men.

### Limitations

There are two main limitations to this qualitative study. Focus group participants were only eligible to take part in the study if they were actively experiencing moderate to severe insomnia symptoms. This characteristic may have biased the discussions to focus on the ways in which social processes worsened insomnia symptoms. Future studies conducted among participants at different stages in their insomnia diagnosis (e.g., acute, chronic, in treatment, and in remission) may yield insights into the positive and negative ways in which social processes influence the lived experience of insomnia. Additionally, because the original study's focus was not to explore the effects of social processes on the lived experience of insomnia, questions specifically probing for this association were not included in the focus group interview guide. The absence of these probing questions may have limited the depth of discussion on this topic. Future studies should incorporate in-depth examinations of participants' social ties and include targeted questions to encourage rich discussions on how social processes impact the lived experience of insomnia.

## Conclusion

This is among the first qualitative study to explore the influence of social processes on the lived experience of insomnia among Latinx adults. The themes brought forth highlight the complex connection between social processes, mainly social stress, social support, and social control and the lived experience of insomnia. These findings suggest that evaluating the social environment of Latinx adults with insomnia symptoms and incorporating aspects of interpersonal psychotherapy (de Mello et al., 2005; Cuijpers et al., 2011) to psychological treatments of insomnia may help reduce the negative effects and enhance the positive effects of social ties on insomnia symptomology in this population. Additionally, given that women described how gender roles and gender identity contributed to their insomnia symptoms, tailoring psychological insomnia interventions to address these gender-specific considerations may improve treatment effectiveness and engagement among Latina women. Study findings underscore the importance of evaluating and engaging the social context of Latinx adults with insomnia in order to more holistically address insomnia symptoms in this population.

## Data Availability

The raw data supporting the conclusions of this article will be made available by the authors, without undue reservation.
