# Supplementary material for: Exploring the social context of insomnia: a thematic content analysis of the lived experiences of insomnia of Latinx women and men
Source: Front Sleep. 2024 Sep 11;3:1456045. doi: 10.3389/frsle.2024.1456045 (PMC12713941; doi:10.3389/frsle.2024.1456045)
Supplement: Supplementary file 1 [file Table_1.DOCX]

Supplementary Materials

**Supplementary Materials A.** Codebook used to Examine the Influence of Social Processes on the Lived Experience of Insomnia among Latinx adults

| Parent nodes | Child nodes | |
| --- | --- | --- |
| Predisposing factors of insomnia | Social support |  |
|  | Social stress |  |
|  | Social Control |  |
|  | Personal Control |  |
|  | Symbolic meaning |  |
|  | Age |  |
| Precipitating factors of insomnia | Social support | Physical health conditions |
|  | Social stress | Shift work |
|  | Social Control | Mental health and emotional issues |
|  | Personal Control | Impactful live events |
|  | Symbolic meaning | Immigration |
| Perpetuating factors of insomnia | Social support | Physical health conditions |
|  | Social stress | Mental health and emotional issues |
|  | Social Control | Lifestyle changes |
|  | Personal Control | Immigrant experiences |
|  | Symbolic meaning | Environmental factors |
|  | Rumination | Economic concerns |
|  | Poor sleep hygiene |  |
| Influential Social Relationships and Contexts | Family | Pets |
|  | Friends | Other |
|  | Spouse or romantic partner |  |
|  | Work colleagues or Work environment |  |
|  | Neighbors or Neighborhood |  |
|  |  |  |
| Coping with insomnia | Social support | Prayer |
|  | Social stress | Mental health therapy |
|  | Social control | Medication |
|  | Personal control | Distractions |
|  | Symbolic meaning | Complementary alternative medicine |
| Insomnia effects on Social relationships | Social support |  |
|  | Social stress |  |
|  | Social control |  |
|  | Personal control |  |
|  | Symbolic meaning |  |
